# Association of teleworking and smoking behavior of U.S. wage and salary workers

**DOI:** 10.1002/1348-9585.12283

**Published:** 2021-10-02

**Authors:** Nigar Nargis, Qing Li, Lauren Griffin, Samuel Asare, Priti Bandi, Anuja Majmundar, J Lee Westmaas, Ahmedin Jemal

**Affiliations:** ^1^ American Cancer Society Atlanta Georgia USA; ^2^ Viametric Solutions LLC Atlanta Georgia USA; ^3^ Emory University Atlanta Georgia USA

**Keywords:** COVID‐19 pandemic, smoking intensity, smoking prevalence, telecommuting

## Abstract

**Introduction:**

The COVID‐19 pandemic has led to a major shift in workspace from office to home. This report examined how telecommuting is related to smoking behavior of wage and salary workers.

**Methods:**

Self‐reported smoking behavior of 1,390 U.S. wage and salary workers aged 16–64 years from the Tobacco Use Supplement of the Current Population Survey 2018/19 were linked to the 2018 American Time Use Survey. Weighted multivariate logistic regression predicting smoking probability and generalized linear regression predicting smoking intensity were used for analysis.

**Results:**

Almost a fifth (19%) of wage and salary workers reported working from home and over a half (52%) reported working in telecommuting amenable occupations. Nearly 12% were current smokers, smoking 14.7 cigarettes daily on average. Compared to their counterparts, smoking prevalence (percentage points) was lower among those employed in telecommuting amenable occupations (−0.52, *p* < .001 for all; 0.01, *p* = .862 for men; −2.40, *p* < .001 for women) and who worked more frequently from home (−0.21, *p* < .001 for all; −0.76, *p* < .001 for men; −0.03, *p* = .045 for women). Smoking intensity (cigarettes per day) was lower among those employed in telecommuting amenable occupations (−3.39, *p* = .03 for all; −0.36, *p* = .90 for men; −4.30, *p* = .21 for women). We found no statistically significant association between smoking intensity and telecommuting frequency.

**Conclusions:**

The lower likelihood of smoking and lower level of smoking intensity among telecommuting wage and salary workers suggests the need for proactive efforts to address the potential exacerbation in occupation‐related smoking disparities between occupations that are and are not amenable to telecommuting.

## 1 INTRODUCTION

There has been a stark shift in the workspace from office to home in the U.S. to prevent the spread of the coronavirus disease 2019 (COVID‐19) pandemic.^1^, ^2^ While this shift may have temporarily affected a select group of employed people, telecommuting may become the norm after the public health crisis is mitigated, leaving a more permanent impact on work‐related behavior of individuals and communities. This is in view of many employers considering work‐from‐home arrangements for their employees permanently, with a majority of teleworkers preferring to work from home.^3^, ^4^, ^5^

The scope of occupational sectors offering work from home arrangements typically include management, education, computer, finance, and law, and exclude farm, construction and production. Recent studies examining the changes in smoking behavior following the onset of the COVID‐19 pandemic in Belgium, Italy, Japan and the United Kingdom observed that people who work from home experienced increased smoking prevalence during the pandemic.^6^, ^7^, ^8^, ^9^ While a few studies in the U.S. have investigated the changes in tobacco use during the pandemic compared to the pre‐pandemic period, none of them examined the changes in smoking behavior specifically among telecommuters.^10^, ^11^, ^12^, ^13^, ^14^, ^15^ Only one previous study, limited to a non‐representative sample of U.S. adults from 2010 to 2011, examined change in smoking behavior, but found greater risk of tobacco use among *non*‐telecommuters in the U.S.^16^

It can be hypothesized that concerns about secondhand or thirdhand smoke exposure and/or residential smoke‐free policies may promote cessation or reduced consumption among smokers who work from home. Conversely, the absence of workplace smoke‐free policies at home may potentially lead to smoking initiation, relapse, or increased consumption. Increased consumption or reduced cessation associated with telecommuting would add to potential increases in smoking triggered by COVID‐19 when individuals may have used smoking as a coping mechanism, or stockpiled cigarettes in fear of supply bottlenecks.^17^ The net effect of working from home on smoking behavior is dependent on the relative strength of the smoke‐free environments at both workplace and home experienced by individual workers.

In this paper, we examined whether telecommuting is associated with smoking behavior among U.S. wage and salary workers based on a national level survey from the pre‐COVID‐19 pandemic period. Findings may inform evidence‐based public health interventions aimed at reducing smoking disparities and organizational health policies focused on employee well‐being in the post‐pandemic era.

## 2 METHODS

In a cross‐sectional study design, self‐reported responses from U.S. wage and salary workers (aged 16–64 years) were used from the Tobacco Use Supplement of the Current Population Survey (TUS‐CPS) July 2018 wave linked to the Leave and Job Flexibilities Module of the 2018 American Time Use Survey (ATUS), matched on household and individual identifiers. The following two‐step model of smoking prevalence and intensity was used for estimation:

(1) $$\ln\frac{SP_{i}}{1‐SP_{i}}=\alpha_{1}X_{i}+\beta_{1}W_{i}+Z_{i}^{\prime }\gamma_{1}+e_{1i}$$

(2) $$SI_{i}=\alpha_{2}X_{i}+\beta_{2}W_{i}+Z_{i}^{\prime }\gamma_{2}+e_{2i}$$

where the outcome variables are smoking probability, *SP*_i_ =1 if worker *i* smoked at least 100 cigarettes in their lifetime and currently smokes every day or somedays, and 0 otherwise; and smoking intensity, *SI*_i_ = average number of cigarettes smoked per day by a current smoker *i*.

The exposure variable, telecommuting frequency, *X*_i_, was coded ‘0’, indicating unable to work from home and 1–4 representing working from home ‘less than once a month’, ‘once a month’, ‘once every two weeks’, and ‘at least once a week’, respectively. We included an occupation variable, *W*_i_, to differentiate the ‘structural zeros’ in *X*_i_ due to the nature of occupations that are typically not amenable to telecommuting (e.g., services, sales, office and administrative support, farming, construction) from the ‘random zeros’ in occupations that are amenable to telecommuting but for which employees did not report working from home during the survey period (e.g., management, business, finance, professional occupations).^18^, ^19^ *W*_i_ was coded ‘0’ if an employee's occupation belonged to the categories typically not amenable to telecommuting (more than 80% respondents in these occupations reported in the survey not being able to work from home) and 1 if an employee's occupation belonged to the categories amenable to telecommuting. The sample statistics by employees’ major occupation groups and telecommuting frequency are provided in Table A1 in the Supplementary File.

The coefficients *β*_1_ and *β*_2_ represent trait effect of telecommuting on the responses, *SP* and *SI*, all other things being equal, while the coefficients *α*_1_ and *α*_2_ measure the changes in responses per unit increase in *X*_i_ (telecommuting frequency) within the group whose jobs are amenable to telecommuting.^20^ The association between telecommuting status and smoking outcomes was tested using a composite linear hypothesis, H_01_:*α*_1_ = 0, *β*_1_ = 0 for smoking prevalence and H_02_:*α*_2_ = 0, *β*_2_ = 0 for smoking intensity. Statistical tests were 2‐sided and considered significant at *α* = 0.10 due to the small sample size of the study, and the fact that, in small samples, meaningful results may fail to appear statistically significant at the conventional level of significance of 0.01 or 0.05.^21^

**FIGURE 1:**
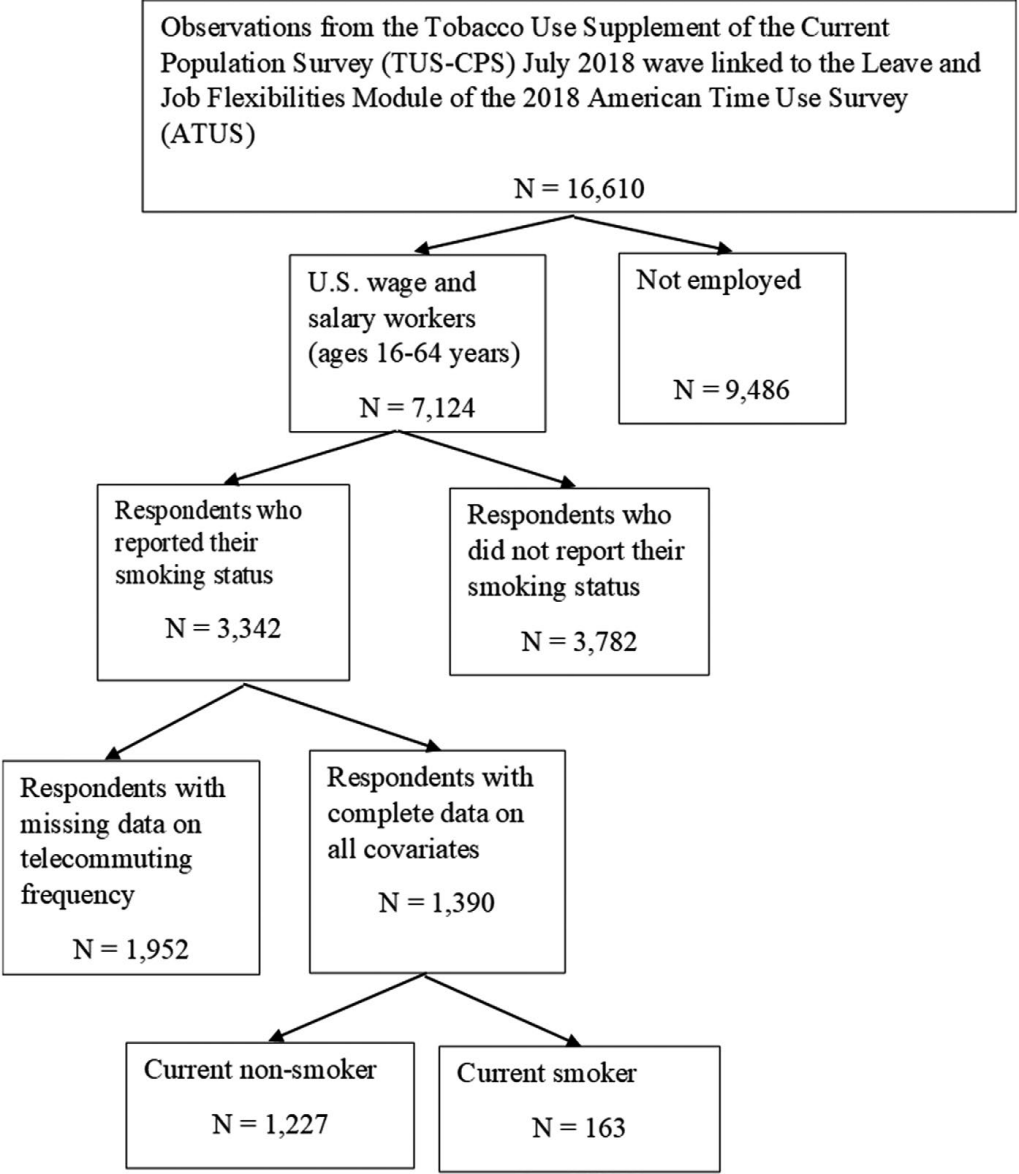
1 Study population and sample selection

Covariates *Z*_i_ included participants’ work status (part‐time, full time, hours vary), socio‐demographic characteristics (e.g., age, race/ethnicity, marital status, presence of children ages 0–5 in the household, annual family income, educational status), and regions from the CPS; smoke‐free air policies from the State Tobacco Activities Tracking and Evaluation (STATE) System; and average cigarette price per pack (dollars) from the Tax Burden on Tobacco database corresponding to the states of residence.^22^, ^23^ The random error terms in the two equations are represented by *e*_1i_ and *e*_2i_. Equations (1) and (2) were estimated using multivariable logit regression and generalized linear regression respectively. The regressions were weighted to be generalizable to the study population and to account for complex sampling design using replicate weights from the CPS (in STATA Version 15). Analyses were stratified by sex due to differences in preference for workplace flexibility between men and women employees.^24^ The analytical sample with non‐missing values for all variables used in the analysis comprised 1,390 employees (690 men and 700 women). The study population and the stages of sample selection are shown in Figure 1.

## 3 RESULTS

Nearly 12% of respondents were current smokers and smoked 14.6 cigarettes per day on average. The prevalence of smoking was higher among men (12.6%) than among women (10.9%). The intensity of smoking among those who were smokers was at the same level of 14.6 cigarettes per day among men and women. Although half of the employees (51.7% overall; 48.4% among men; 54.9% among women) were employed in occupations that are amenable to telecommuting, only 19.2% (18.0% among men; 20.1% among women) reported working from home in varied frequencies ranging from less than once a month to at least once a week. Two‐thirds of the employees were full‐time workers, about a tenth were part‐time workers, and a quarter reported varying work hours. The summary statistics of all the covariates including demographic characteristics, socio‐economic status, state‐level tobacco control policy status and region of residence of respondents are provided in Table 1.

**TABLE 1:** Sample characteristics of U.S. wage and salary workers ages 16–64, 2018

Characteristics	All		Men		Women	
	Mean	[95%–CI]	Mean	[95%–CI]	Mean	[95%–CI]
Smoking outcomes						
Smoking prevalence (%)	11.7	10.0–13.4	12.6	10.1–15.1	10.9	8.5–13.2
Smoking intensity (number of cigarettes smoked daily)	14.6	13.3–15.9	14.6	12.8–16.4	14.6	12.6–16.5
Telecommuting frequency (%)						
0 ‐ None	80.9	78.8–82.9	82.0	79.2–84.9	79.7	76.7–82.7
1 ‐ Less than once a month	3.1	2.2–4.0	3.8	2.3–5.2	2.4	1.3–3.6
2 ‐ Once a month	2.9	2.0–3.8	3.6	2.2–5.0	2.1	1.1–3.2
3 ‐ Once every 2 weeks	3.1	2.2–4.0	2.9	1.6–4.2	3.3	2.0–4.6
4 ‐ At least once a week	10.1	8.5–11.7	7.7	5.7–9.7	12.4	10.0–14.9
Total	100.0		100.0		100.0	
Occupations amenable to telecommuting (%)	51.7	49.0–54.3	48.4	44.7–52.1	54.9	51.2–58.6
Work status (%)						
Full‐time	66.1	63.6–68.6	66.4	62.8–69.9	65.8	62.3–69.4
Part‐time	9.1	7.5–10.6	8.1	6.1–10.1	10.0	7.8–12.2
Hours vary	24.8	22.5–27.1	25.5	22.2–28.8	24.1	21.0–27.3
Total	100.0		100.0		100.0	
Women (%)	50.3	47.7–53.0	‐	‐–‐	‐	‐–‐
Age (years)	43.3	42.7–44.0	42.6	41.7–43.5	44.0	43.1–44.9
Presence of children (ages 0–5) in household (%)	14.9	13.0–16.8	16.1	13.3–18.8	13.7	11.2–16.3
Race/ethnicity (%)						
White, non‐Hispanic	68.8	66.4–71.3	69.0	65.5–72.4	68.7	65.3–72.1
Black, non‐Hispanic	11.6	10.0–13.3	10.0	7.7–12.2	13.3	10.8–15.8
Hispanic	12.9	11.1–14.6	12.9	10.4–15.4	12.8	10.4–15.3
Other	6.6	5.3–7.9	8.1	6.1–10.1	5.1	3.5–6.8
Total	100.0		100.0		100.0	
Marital status (%)						
Married	46.7	44.1–49.3	51.0	47.3–54.8	42.4	38.8–46.1
Widowed	2.3	1.5–3.1	1.4	0.6–2.3	3.1	1.8–4.4
Divorced/separated	18.9	16.9–21.0	14.3	11.7–17.0	23.4	20.3–26.6
Never married	32.1	29.6–34.5	33.2	29.7–36.7	31.0	27.6–34.4
Total	100.0		100.0		100.0	
Educational status (%)						
Less than high school	6.8	5.4–8.1	7.2	5.3–9.2	6.3	4.5–8.1
High school or equivalent	23.5	21.2–25.7	23.3	20.1–26.5	23.6	20.4–26.7
Some college education	28.3	26.0–30.7	29.7	26.3–33.1	27.0	23.7–30.3
College graduate	25.0	22.8–27.3	23.3	20.2–26.5	26.7	23.4–30.0
More than college	16.4	14.4–18.4	16.4	13.6–19.1	16.4	13.7–19.2
Total	100.0		100.0		100.0	
Annual family income in relation to federal income poverty line (FIPL) (%)						
Below 100% of FIPL	5.5	4.3–6.7	5.5	3.8–7.2	5.4	3.7–7.1
100% to 200% of FIPL	11.5	9.8–13.2	11.2	8.8–13.5	11.9	9.5–14.3
200% to 400% of FIPL	28.3	25.9–30.6	31.2	27.7–34.6	25.4	22.2–28.7
Above 400% FIPL	54.7	52.1–57.4	52.2	48.4–55.9	57.3	53.6–61.0
Total	100.0		100.0		100.0	
Tobacco control policy variables						
Average cigarette price per pack ($)	7.21	7.12–7.28	7.22	7.11–7.34	7.19	7.07–7.30
Smoking ban in						
Government worksite (%)	78.8	76.7–81.0	78.6	75.4–81.6	79.1	76.1–82.2
Private worksite (%)	73.1	70.8–75.4	73.2	69.9–76.5	73.0	69.7–76.3
Restaurant (%)	68.6	66.2–71.1	69.0	65.5–72.4	68.3	64.8–71.7
Bars (%)	58.0	55.4–60.6	60.0	56.2–63.5	56.1	52.4–59.8
Region (%)						
Northwest	16.3	14.4–18.3	16.8	14.0–19.6	15.9	13.1–18.6
Midwest	25.8	23.5–28.1	25.8	22.5–29.1	25.7	22.5–29.0
South	35.0	32.4–37.5	34.3	30.8–37.9	35.6	32.0–39.1
West	22.9	20.7–25.2	23.0	19.9–26.2	22.9	19.7–26.0
Total	100.0		100.0		100.0	
Number of observations	1390		690		700	

Note

The mean and 95% CI of smoking intensity are based on responses from 105 current smokers. The sum of percentages of groups for each variable may not be exactly 100% due to rounding‐off of individual group percentages. The percentages for the smoking ban variables for government worksite, private worksite, restaurant, and bar would not add up to 100% because these characteristics of tobacco control policy status of states are not mutually exclusive. One state can have smoking ban in one or more sites.

Abbreviation: CI, confidence interval.

Smoking probability was negatively associated with employment in telecommuting amenable occupations, but in stratified analyses was significant among women but not men. The overall negative association was stronger with a higher frequency of telecommuting (Table 2). On average, the probability of smoking by an employee in a telecommuting amenable occupation was 0.52 percentage points lower than those in occupations not amenable to telecommuting and the probability decreased by 0.21 percentage points for each unit increase in telecommuting frequency. The combined effect of occupation and telecommuting frequency on smoking prevalence was negative and grew stronger in a dose‐response fashion with a higher frequency of telecommuting (from −0.73 to −1.36 percentage points). Similar negative associations of smoking status with telecommuting frequency, employment in telecommuting amenable occupation, and the combined effects were found in women employees. Among men, the dose response negative association was observed between smoking status and telecommuting frequency, while the association with employment in telecommuting amenable occupations was not statistically significant.

**TABLE 2:** Estimates of the association of smoking prevalence and smoking intensity with telecommuting status of U.S. wage and salary workers ages 16–64

Smoking prevalence	All			Smoking prevalence, % (N)	Men		Smoking prevalence, % (N)	Women	
	Smoking prevalence, % (N)	Marginal effect, % points (95% CI)	*p*		Marginal effect, % points (95% CI)	*p*		Marginal effect, % points (95% CI)	*p*
Telecommuting frequency		**−0.21** (−0.23, −0.18)	<.001		**−0.76** (−0.80, −0.72)	<.001		**−0.03** (−0.06, −0.00)	.045
Employment in telecommuting amenable occupation		**−0.52** (−0.58, −0.45)	<.001		0.01 (−0.09, 0.11)	0.862		**−2.40** (−2.48, −2.31)	<.001
Combined effect									
0 – None	12.0% (1124)			12.9% (566)			11.1% (558)		
1 – Less than once a month (%)	23.2% (43)	−0.73		23.1% (26)	−0.76		23.5% (17)	−2.43	
2 – Once a month (%)	7.5% (40)	−0.94		8.0% (25)	−1.52		6.7% (15)	−2.46	
3 – Once every 2 weeks (%)	11.6% (43)	−1.15		10.0% (20)	−2.28		13.0% (23)	−2.49	
4 – At least once a week (%)	7.1% (140)	−1.36		7.5% (53)	−3.04		6.9% (87)	−2.52	
Joint test of marginal effects of telecommuting frequency and employment in telecommuting amenable occupations = 0		Chi‐square(2) = 734.05	<.001		Chi‐square(2) = 1493.52	<.001		Chi‐square(2) = 3995.75	<.001
Smoking intensity									
Telecommuting frequency		0.48 (−0.84, 1.80)	0.48		2.02 (−0.36, 4.42)	0.10		1.88 (−0.82, 4.59)	0.17
Employment in telecommuting amenable occupation		−3.39 (−6.38, −0.41)	0.03		−0.36 (−5.79, 5.07)	0.90		−4.30 (−9.63, 1.03)	0.11
Combined effect		−3.39			‐			‐	
Joint test of marginal effects of telecommuting frequency and employment in telecommuting amenable occupations =0		Chi‐square (2) = 5.11	0.08		Chi‐square (2) = 2.89	0.24		Chi‐square (2) = 3.13	0.21

Note

The logit regression for smoking prevalence and the generalized linear regression for intensity controlled for full‐time/part‐time work status, sex, age, presence of children (ages 0–5) in household, race/ethnicity, marital status, educational status, annual family income in relation to the federal income poverty line, tobacco control policy variables, and region of residence.

Among those who smoked, smoking intensity was negatively associated with employment in telecommuting amenable occupations. The association did not differ by telecommuting frequency. A similar negative association between smoking intensity and employment in telecommuting amenable occupations was observed among men and women employees. The estimates were, however, not statistically significant.

## 4 DISCUSSION

Based on a national level contemporary database, this study found that smoking probability was negatively associated with employment in telecommuting amenable occupations and telecommuting frequency, except for men employees who demonstrated a negative association for telecommuting frequency only. These effects were obtained after controlling for part/full‐time work status, education, income, other sociodemographic and tobacco control policy and geographic variables. It is possible that individuals who work from home are less likely to smoke due to smoke‐free housing laws and concerns for non‐smoking family members’ secondhand smoke exposure.

The negative relationship of employment in telecommuting amenable occupations with women's smoking status is far stronger than in overall population. This finding is supported by a nationally representative study on working women of reproductive age (18–49 years) in the U.S. for the period 2009–2013. Based on the number of women workers and smoking prevalence classified by occupation in this study, nearly two‐thirds women workers were employed in *non*‐telecommuting amenable occupations with median smoking prevalence of 21.1% in contrast to 12.4% median smoking prevalence among those employed in telecommuting amenable occupations.^25^ The overall negative association of smoking prevalence and telecommuting status suggests the potential effectiveness of strengthening the implementation of smoke‐free housing laws and building public awareness about the harmful health effects of secondhand and thirdhand smoke exposure.

Given lower smoking prevalence and intensity are associated with lower risk of smoking‐related morbidity and mortality,^26^, ^27^ if the negative association between smoking prevalence and intensity with telecommuting status prevails during the pandemic, working‐from‐home may have positive external effects on smoking behavior and health outcomes. These health benefits may, however, accrue unevenly to those engaged in telecommuting amenable occupations. Given that nearly half of the employees in this sample worked in occupations not amenable to telecommuting, it is possible that the large‐scale shift to work from home post‐pandemic may exacerbate occupation‐related smoking inequalities. Pro‐active efforts to address these potential occupational smoking disparities are needed, including implementation and strengthening of tobacco control efforts (smoke‐free workplaces; workplace cessation programs) among occupations not amenable to telecommuting.

This study has a few limitations. First, the point estimates for the associations between smoking intensity and telecommuting had wide confidence intervals as they were based on a small sample size (total of 105 participants). Therefore, the findings should be interpreted cautiously.

Second, the findings in this paper may not generalize to the self‐employed population as the Leave and Job Flexibilities Module of the 2018 ATUS data did not include the self‐employed. Finally, 5,734 wage and salary workers with missing values on smoking status (*N* = 3782) and telecommuting frequency (*N* = 1952) were excluded from the final analysis (as shown in Figure 1). These individuals were of disproportionately lower education status (42.6% with less than high school diploma vs. 6.8% of the analytical sample), which can be a source of selection bias. However, the use of the dummy variable indicating occupations amenable or not amenable to telecommuting potentially corrects for this bias as the observations missing telecommuting frequency are inherently not amenable to telecommuting. As the missingness of only telecommuting frequency among all covariates is not random, the use of multiple imputation method for the incomplete cases would have introduced further selection bias.^28^ This study, therefore, relies on complete case analysis by excluding all incomplete cases.

The generalizability of the results of this study based on pre‐pandemic data to the post‐pandemic scenario is limited by the unavailability of real time data from the post‐COVID‐19‐onset period. First, the reduction in smoking probability with increasing frequency of teleworking, as observed in this study, might be partly driven by lower productivity of workers who work more frequently from home, lower income and purchasing power and hence reduced demand for cigarettes. This effect is expected to be more pronounced immediately after a sudden shock, such as switching from 100% or partial office work to 100% telework that took place as part of the pandemic response. At the same time, working from home reduced the risk of exposure to the virus, reduced the probability of illness and deaths and in turn protected earning household members from loss of productivity in the medium to longer term. As the current analysis used data from a pre‐pandemic cross‐sectional survey, it was not feasible to identify this productivity response mechanism.

Second, based on occupational classification by the feasibility of working from home, previous research identified that 37% of jobs in the U.S. could plausibly be performed at home and these jobs typically pay more than the jobs that cannot be performed from home accounting for 46% of all U.S. wages.^18^ Individuals who cannot work from home are more likely to be lower‐income, have less than college education, reside in rental housing, be non‐white, and lack employer‐provided health insurance.^19^ The negative association between smoking prevalence and the frequency of working from home observed in this study from the pre‐pandemic period may, therefore, be driven by the underlying socio‐economic disparity in smoking behavior.

Third, the frequency of working from home in the pre‐pandemic period is a choice variable for the wage and salary workers who could telecommute in their current employment. If there is a common unobserved trait among respondents that led them to decide not to smoke and work from home more frequently, the estimated coefficient representing the association between these two choice variables would suffer from simultaneity bias. This is in sharp contrast with the circumstances in the post‐COVID‐19‐onset period when the work from home order was exogenously imposed on all as an emergency response and the coefficient of the work from home variable would identify the true effect of working from home on smoking behavior.

Finally, working from home has been applicable to all wage and salary workers who are able to work from home in response to the COVID‐19 pandemic irrespective of whether they are able to do part or all of their work at home. In a study based on real‐time measures of work from home during April‐May 2020, nearly half of survey respondents reported working from home, including 35.2% who recently switched from commuting to working from home.^29^ A similar study observed that 8.2% of the U.S. workforce were working from home in February 2020 and this percentage went up to 35.2% in May 2020.^2^ If a home‐based work environment is conducive to reducing the odds of smoking, it can be expected that the new order of exclusively working from home would induce lower smoking prevalence among employees who are employed in the sectors subject to remote work arrangements.

## 5 CONCLUSIONS

Pre‐COVID‐19 pandemic, there is a lower likelihood of smoking in telecommuting amenable occupations and with higher telecommuting frequency among U.S. wage and salary workers. Smoking intensity is also lower in telecommuting amenable occupations. These findings suggest the need for proactive efforts to address the potential exacerbation in occupation‐related smoking disparities between occupations that are and are not amenable to telecommuting.

## DISCLOSURES

*Ethical approval*: N/A. *Informed consent*: N/A. *Registry and the Registration No*. *of the study*/*Trial*: N/A. *Animal Studies*: N/A. *Conflict of interest*: None.

## AUTHOR CONTRIBUTIONS

Nigar Nargis: Writing‐Original draft preparation, Methodology. Qing Li: Conceptualization, Data curation, Visualization, Investigation. Lauren Griffin: Formal analysis, Software. Samuel Asare: Writing‐Reviewing and Editing, Validation. Priti Bandi: Writing‐Reviewing and Editing, Validation. Anuja Majmundar: Writing‐Reviewing and Editing, Validation. J Lee Westmaas: Writing‐Reviewing and Editing. Ahmedin Jemal: Writing‐Reviewing and Editing, Supervision.

## Supporting information

Supplementary Material
