# Supplementary material for: Association of teleworking and smoking behavior of U.S. wage and salary workers
Source: J Occup Health. 2021 Oct 2;63(1):e12283. doi: 10.1002/1348-9585.12283 (PMC8487165; doi:10.1002/1348-9585.12283)
Supplement: Supplementary file 1 — Supplementary Material [file JOH2-63-e12283-s001.pdf]

Table A1: Telecommuting frequency of U.S. wage and salary workers ages 16-64 by major occupation groups, 2018.

Major Occupation Groups		Telecommuting frequency					
		None	Less than once a month	Once a month	Once every 2 weeks	At least once a week	Total
**ALL (n=1,390 workers)**							
Management, business, and finance	N (%)	179 (67.0)	17 (6.4)	15 (5.6)	16 (6.0)	40 (15.0)	267 (100.0)
Professional and related work	N (%)	311 (69.0)	21 (4.7)	19 (4.2)	21 (4.7)	79 (7.5)	451 (100.0)
Service	N (%)	168 (97.7)	1 (0.6)	0 (0.0)	0 (0.0)	3 (1.7)	172 (100.0)
Sales and related work	N (%)	81 (83.5)	2 (2.1)	3 (3.1)	2 (2.1)	9 (9.3)	97 (100.0)
Office and administrative support	N (%)	148 (90.8)	0 (0.0)	2 (1.2)	4 (2.4)	9 (5.5)	163 (100.0)
Farming, fishing, and forestry	N (%)	9 (100.0)	0 (0.0)	0 (0.0)	0 (0.0)	0 (0.0)	9 (100.0)
Construction and extraction	N (%)	43 (95.6)	1 (2.2)	1 (2.2)	0 (0.0)	0 (0.0)	45 (100.0)
Installation, maintenance, and repair	N (%)	35 (100.0)	0 (0.0)	0 (0.0)	0 (0.0)	0 (0.0)	35 (100.0)
Production	N (%)	76 (98.7)	1 (1.3)	0 (0.0)	0 (0.0)	0 (0.0)	77 (100.0)
Transportation and material moving	N (%)	74 (100.0)	0 (0.0)	0 (0.0)	0 (0.0)	0 (0.0)	74 (100.0)
**MEN (n=690 workers)**							
Management, business, and finance	N (%)	90 (68.7)	8 (6.1)	11 (8.4)	9 (6.9)	13 (9.9)	131 (100.0)
Professional and related work	N (%)	133 (65.5)	14 (6.9)	13 (6.4)	10 (4.9)	33 (16.3)	203 (100.0)
Service	N (%)	67 (94.4)	1 (1.4)	0 (0.0)	0 (0.0)	3 (4.2)	71 (100.0)
Sales and related work	N (%)	39 (90.7)	1 (2.3)	0 (0.0)	1 (2.3)	2 (4.6)	43 (100.0)
Office and administrative support	N (%)	46 (95.8)	0 (0.0)	0 (0.0)	0 (0.0)	2 (4.2)	48 (100.0)

Farming, fishing, and forestry	N (%)	7 (100.0)	0 (0.0)	0 (0.0)	0 (0.0)	0 (0.0)	7 (100.0)
Construction and extraction	N (%)	41 (95.3)	1 (2.3)	1 (2.3)	0 (0.0)	0 (0.0)	42 (100.0)
Installation, maintenance, and repair	N (%)	33 (100.0)	0 (0.0)	0 (0.0)	0 (0.0)	0 (0.0)	33 (100.0)
Production	N (%)	45 (97.8)	1 (2.2)	0 (0.0)	0 (0.0)	0 (0.0)	46 (100.0)
Transportation and material moving	N (%)	65 (100.0)	0 (0.0)	0 (0.0)	0 (0.0)	0 (0.0)	65 (100.0)
**WOMEN (n=700 workers)**							
Management, business, and finance	N (%)	89 (65.4)	9 (6.6)	4 (2.9)	7 (5.1)	27 (19.8)	136 (100.0)
Professional and related work	N (%)	178 (71.2)	7 (2.8)	6 (2.4)	11 (4.4)	46 (18.5)	248 (100.0)
Service	N (%)	101 (100.0)	0 (0.0)	0 (0.0)	0 (0.0)	0 (0.0)	101 (100.0)
Sales and related work	N (%)	42 (77.8)	1 (1.8)	3 (5.6)	1 (1.8)	7 (13.0)	54 (100.0)
Office and administrative support	N (%)	102 (88.7)	0 (0.0)	2 (1.7)	4 (3.5)	7 (6.1)	115 (100.0)
Farming, fishing, and forestry	N (%)	2 (100.0)	0 (0.0)	0 (0.0)	0 (0.0)	0 (0.0)	2 (100.0)
Construction and extraction	N (%)	2 (100.0)	0 (0.0)	0 (0.0)	0 (0.0)	0 (0.0)	2 (100.0)
Installation, maintenance, and repair	N (%)	2 (100.0)	0 (0.0)	0 (0.0)	0 (0.0)	0 (0.0)	2 (100.0)
Production	N (%)	31 (100.0)	0 (0.0)	0 (0.0)	0 (0.0)	0 (0.0)	31 (100.0)
Transportation and material moving	N (%)	9 (100.0)	0 (0.0)	0 (0.0)	0 (0.0)	0 (0.0)	9 (100.0)

Notes:

1. N = Number of observations. Row percentages are in parentheses below N.

2. The major occupation groups were drawn from the Current Population Survey that follows the U.S. Census Bureau 2018 occupation classification system. The description of each major occupation group is available at:

https://www2.census.gov/programs-surveys/cps/methodology/Occupation%20Codes.pdf
